# Decision coaching using a patient decision aid for youth and parents considering insulin delivery methods for type 1 diabetes: a pre/post study

**DOI:** 10.1186/s12887-019-1898-4

**Published:** 2020-01-03

**Authors:** Margaret L. Lawson, Allyson L. Shephard, Bryan Feenstra, Laura Boland, Nadia Sourial, Dawn Stacey

**Affiliations:** 1 Division of Endocrinology and Metabolism, Children’s Hospital of Eastern Ontario, University of Ottawa, 401 Smyth Road, Ottawa, Ontario K1H 8L1 Canada; 2 Children’s Hospital of Eastern Ontario (CHEO), 401 Smyth Road, Ottawa, ON K1H 8L1 Canada; 3 University of Ottawa, School of Nursing, 451 Smyth Rd, Ottawa, ON K1H 8M5 Canada; 4 University of Ottawa, Population Health, 25 University Private, Ottawa, ON K1N 7K4 Canada

**Keywords:** Shared decision making, Decision coaching, Patient decision aid, Decisional conflict, Type I diabetes

## Abstract

**Background:**

Choice of insulin delivery for type 1 diabetes can be difficult for many parents and children. We evaluated decision coaching using a patient decision aid for helping youth with type 1 diabetes and parents decide about insulin delivery method.

**Methods:**

A pre/post design. Youth and parent(s) attending a pediatric diabetes clinic in a tertiary care centre were referred to the intervention by their pediatric endocrinologist or diabetes physician between September 2013 and May 2015. A decision coach guided youth and their parents in completing a patient decision aid that was pre-populated with evidence on insulin delivery options. Primary outcomes were youth and parent scores on the low literary version of the validated Decisional Conflict Scale (DCS).

**Results:**

Forty-five youth (mean age = 12.5 ± 2.9 years) and 66 parents (45.8 ± 5.6 years) participated. From pre- to post-intervention, youth and parent decisional conflict decreased significantly (youth mean DCS score was 32.0 vs 6.6, *p* < 0.0001; parent 37.6 vs 3.5, *p* < 0.0001). Youth’s and parents’ mean decisional conflict scores were also significantly improved for DCS subscales (informed, values clarity, support, and certainty). 92% of youth and 94% of parents were satisfied with the decision coaching and patient decision aid. Coaching sessions averaged 55 min. Parents (90%) reported that the session was the right length of time; some youth (16%) reported that it was too long.

**Conclusion:**

Decision coaching with a patient decision aid reduced decisional conflict for youth and parents facing a decision about insulin delivery method.

## Background

Youth with type 1 diabetes (T1D) have several options for their insulin delivery method including standard therapy or an intensified regimen with multiple daily injections or insulin pump therapy [1, 2]. As such, the choice of insulin delivery method is *preference sensitive*, regardless of the youths’ age, requiring the potential benefits and harms of each option to be weighed by the patient and their family when deciding a treatment plan. The American Academy of Pediatrics and the Canadian Pediatric Society recommend that healthcare professionals collaborate with youth and parents in decisions about their care, regardless of age [3, 4]. Shared decision making involves patients and families together with clinicians discussing the best available evidence about options and patients’ informed preferences to make the best treatment decision for the child [5]. Although, shared decision making appears to have a positive effect on diabetes outcomes [6], pediatric healthcare professionals, patients, and families experience barriers to engaging in shared decision making [7]. Patient decision aids and decision coaching are effective interventions for facilitating shared decision making [8, 9]. Patient decision aids are clinical tools that make explicit the decision, provide information on options, benefits, and harms, and help clarify patients’ values associated with outcomes of options. Decision coaching is non-directive guidance to prepare the patient and family to make a health decision with their healthcare provider.

Despite high quality evidence supporting the efficacy and effectiveness of these shared decision making interventions, few have been evaluated for supporting youth engagement in decision making [10, 11]. In 2012, we conducted a pilot study to evaluate the feasibility of delivering a decision support intervention consisting of decision coaching using a patient decision aid with youth (*n* = 7) ages 9 to 17 years who were considering a change in insulin delivery method and their parents or legal guardians (collectively referred to as ‘parents’). We found the intervention was feasible and acceptable to youth and parents [12]. The objective of this study was to evaluate decision coaching using a patient decision aid on youth and parents’ decisional conflict (primary outcome). Decisional conflict is a state of uncertainty about which course of action to take, manifested by verbalized uncertainty, concern over undesired outcomes, vacillation, and decision delay, sometimes accompanied by general emotional distress [13]. We hypothesized that parent and youth decisional conflict would decrease after exposure to the shared decision making intervention.

## Methods

### Study design

We conducted a pre/post study guided by the Ottawa Decision Support Framework (ODSF) [14]. This framework is helpful for understanding a deliberative process for making a preference-sensitive decision, or a decision with more than one option, with unknown outcomes, or the known benefits and harms can be valued differently by patients. The ODSF posits that health decisions are negatively influenced by unmet decisional needs, such as feeling uninformed, having unclear values, and feeling unsupported, and unresolved decisional conflict. Quality decisions are more likely when patients are informed by the best available evidence and the patient’s values and preferences [14, 15]. In short, decisional needs can be addressed using decisional support interventions (decision coaching using a patient decision aid) to improve decisional outcomes and reduce decisional conflict [16]. The study was approved by the Research Ethics Boards at the Children’s Hospital of Eastern Ontario and the University of Ottawa.

### Participants and setting

Youth and their parents were recruited from an ambulatory diabetes program at a tertiary academic pediatric hospital. The hospital serves an urban and rural population of 1.3 million in Eastern Ontario, Canada; the diabetes program provides care for 850 children and youth with T1D. At the time of the study in 2013–2015, MDI was rarely used by children or youth in our centre. Since then, MDI has become the usual insulin delivery method from diagnosis onwards.

We recruited youth and parents who had told either their pediatric endocrinologist or pediatric diabetes physician during their regular diabetes clinic visit that they were considering a change in insulin delivery method, were capable of participating in the decision making process and were scheduled for decision coaching by one of our diabetes social workers which is a step in the process for youth in our clinic who are considering a change in insulin delivery method. To be eligible for this study, youth had to be under 18 years old with type 1 diabetes duration of at least 10 months, and they and their parents had to be able to read and speak English or French. No lower age limit was set for youth participants, as required by our Research Ethics Boards, provided the youth and parent(s) could participate in the consent or assent process. Family dyads (youth and one parent) and family triads (youth and two parents) were included. The study was introduced to youth and parents being scheduled for decision coaching by the administrative assistant for the diabetes team. A research assistant contacted those who expressed interest in the study. This contact was by telephone to assess study eligibility and explain the study in detail. Youth and parents, regardless of the youth’s age, who agreed to participate provided written informed consent, and assent by the youth if necessary, prior to the decision coaching.

### Procedures

Youth and parents were referred for decision coaching by their diabetes physician. Decision coaching sessions were conducted in private meeting rooms within the diabetes clinic and booked separately from regular diabetes clinic follow-up appointments. Data were independently collected from youth and parent participants at three time points: baseline (T1), immediately post decision coaching (T2), and 10 to 14 days post decision coaching (T3) by return mail. At T1 and T2, a research assistant was present to help the youth and parents’ work through the data collection form by explaining the forms and to answer any questions. Participants did not adopt a new insulin delivery method prior to T3.

### Intervention to support shared decision making

Participants received decision coaching guided by the Ottawa Family Decision Guide (OFDG), a generic decision aid suitable for any difficult health or social decision [16]. The OFDG is a practical shared decision making tool that provides a structured approach for multiple stakeholders to work through the decision making process, which was available in both English and French. For this study, the OFDG was pre-populated in advance with evidence on benefits and harms of each of the three insulin delivery options based on the Canadian Diabetes Association Clinical Practice Guidelines (1) and reviewed by the local centre’s pediatric diabetes educators and pediatric endocrinologists. Options included: a) standard insulin therapy (2 or 3 injections/day) at the time of the study, b) multiple daily injections and, c) insulin pump therapy.

Two social workers, who were members of the diabetes team and trained in interprofessional shared decision making and decision coaching, provided tailored decision coaching sessions to family dyads and triads. The diabetes interprofessional team nominated social workers as decision coaches for this study because it was thought that decision coaching was a ‘natural’ fit in the social workers’ role in this clinic and the intervention could be incorporated into the clinical pathway with little disruption. Decision coaching training consisted of the Ottawa Decision Support Tutorial and a 3-h skills building workshop. This training intervention has been shown efficacious for improving healthcare providers’ decision support knowledge in randomized controlled [17]. The social workers followed a decision coaching protocol to guide the sessions, which emphasized encouraging the youth to speak first to minimize power-imbalances and discourage parents’ biasing the youth’s responses.

We made two changes from the pilot study [12], based on feedback from the decision coaches. The first was to change the introductory question from “Should I/my child get an insulin pump?” to “What is the best insulin delivery method for me/my child?” We also changed the order in which the options were presented, so that insulin pump therapy became the third instead of the first option presented in the coaching session. The rationale for these changes was to minimize perceived bias that may have been present in the pilot study related to positioning insulin pump therapy as the first, and therefore potentially preferred option, for consideration.

### Outcome measures

Paper-based, self-administered questionnaires were used to measure participants’ decisional conflict (T1 and T3), choice predisposition (T1 and T2), and satisfaction with the decision coaching intervention (T3). The primary outcome was change in decisional conflict from T1 to T3. We used the low literacy version of the validated Decisional Conflict Scale (DCS) [18, 19]. We chose this version to accommodate various literacy levels when working with youth and parents. The low literacy DCS has good internal reliability with alpha coefficients ranging from 0.80 to 0.86 [18]. The 10-item low literary DCS evaluates four domains of decisional conflict: a) feeling informed (3 items), b) feeling clear about personal values (2 items questions), c) feeling supported in decision making (3 items), and d) feeling certain about best choice (2 items). Each question has three response categories (yes/no/unsure). The scale ranges from 0 (no decisional conflict) to 100 (extremely high decisional conflict). Scores equal to or exceeding 37.5 are associated with delaying decision making or feeling unsure; scores less than 25 are associated with implementing the decision [18].

Secondary outcomes were: change in choice predisposition from T1 to T2, dyad agreement about preferred choice, and satisfaction with the intervention. The validated Choice Predisposition Scale was used to measure the preferred option pre-intervention [20]. This scale has a test-retest coefficient > 0.90 [20, 21]. We compared choice predisposition at T1 to immediately after the intervention (T2) and compared the youth’s choice at T1 and T2 to parent(s). When two parents participated, we included both parents’ responses and compared their choices by randomly choosing 1 parent. Participants’ satisfaction with the intervention was measured using a questionnaire that combined a SDM satisfaction rating tool [22] and a modified version of the Genetic Counselling Satisfaction Scale which has excellent reliability with an alpha coefficient of 0.90 [22, 23].

### Data analysis

We managed our data using REDCap software, a secure web-based data management application [24]. Descriptive statistics were used to summarize demographics outcomes. Means (with standard deviations) were calculated for continuous variables, and proportions for categorical variables. A paired t-test was used to compare the overall DCS score pre (T1) and post coaching (T3). Exploratory t-tests were also conducted for the DCS sub-scales pre and post. Cohen’s kappa coefficient was used to determine rater agreement about the preferred treatment option within each family (i.e., youth-mother, youth-father, mother-father) before and immediately after decision coaching. Overall treatment option agreement was evaluated using the Fleiss’ kappa coefficient, which accounts for agreement between multiple raters (e.g., youth-mother-father). Statistical significance was set at *p* < 0.05 and adjusted for the four DCS subscale analyses to make the significance level 0.0125. With decisional conflict as the primary measure, the sample size of 45 provided 80% power to detect an effect size of 0.4 with alpha of 0.05 allowing for 10% attrition rate.

## Results

Seventy-three percent (45/62) of eligible families consented to participate (Table 1) between September 2013 and May 2015. At baseline, the sample consisted of 45 youth and 66 parents, including 24 dyads (19 youth with their mother and 5 youth with their father) and 21 triads (21 youth with both parents). The youth ranged in age from 6.3 to 17.7 years with T1D duration of 10 months to more than 5 years. All youth were on standard insulin therapy or MDI prior to the decision coaching. Thirty-seven youth (82%) and 51 parents (77%) returned T3 study measures by mail. Twenty-four sessions (53%) were attended by the youth and one parent, with remaining 21 sessions attended by the youth and both parents.

**Table 1:** Demographic Characteristics of Participants

Youth (*n* = 45)	
Age in years - mean (SD)	12.5 (2.9)
Age in years - range	6.3–17.7
Female sex – no. (%)	20 (44.4)
English primary language – no. (%)	44 (97.8)
Duration of T1DM – no. (%)	
< 10 months	0 (0)
10 months – 1 year	17 (37.8)
> 1 year – 5 years	18 (40)
> 5 years	10 (22.2)
Education – no. (%)	
< Grade 4	10 (22.2)
Grades 5–8	18 (40)
Grades 9–12	16 (35.6)
1st year university	1 (2.2)
Parents (*n* = 66)	
Age in years - mean (SD)	45.8 (5.6)
Age in years - range	36–59
Female sex – no. (%)	40 (60.6)
English primary language – no. (%)	61 (92.4)
Marital status – no. (%)	
Married/common law	52 (78.8)
Divorced/separated	12 (18.2)
Single	2 (3)
Relationship to youth – no. (%)	
Mother	37 (56)
Father	25 (37.9)
Legal guardian	4 (6.1)
Education – no. (%)	
High school	14 (21.2)
Trade certificate / diploma	11 (16.7)
College / university diploma	34 (51.5)
Post graduate studies	7 (10.6)

### Decisional conflict score

Youths’ mean total decisional conflict score decreased from 32.0, SD 19.7 at baseline to 6.6, SD 12.2 at T3 (*p* < 0.0001) after decision coaching. Parents’ mean total decisional conflict score decreased from 37.6, SD 20.7 to 3.5, SD 7.4 (*p* < 0.0001). Youth’s and parents’ mean decisional conflict scores were significantly improved for subscales indicating they: felt informed, had values clarity, felt supported, and were sure of their decision (Table 2).

**Table 2:** Change in participants’ decisional conflict scores post decision coaching

Decisional Conflict Scale	Baseline (T1) Mean ± SD	Post (T3) Mean ± SD	Mean change	*P* value
Youth (*n* = 37)				
Total DCS^1^	32.0 ± 19.7	6.6 ± 12.2	−25.4	< 0.0001
Subscales				
Informed	51.8 ± 26.9	9.0 ± 17.8	− 42.8	< 0.0001
Values clarity	48.6 ± 33.8	6.1 ± 18.1	−42.5	< 0.0001
Support	20.7 ± 18.6	3.2 ± 8.6	−17.5	< 0.0001
Certainty	35.8 ± 32.6	8.8 ± 19.7	−27.0	< 0.0001
Parents (*n* = 53)				
Total DCS^1^	37.6 ± 20.7	3.5 ± 7.4	−34.1	< 0.0001
Subscales				
Informed	52.6 ± 30.5	2.9 ± 9.2	−49.7	< 0.0001
Values clarity	44.7 ± 34.1	0.0 ± 0.0	−44.7	< 0.0001
Support	23.9 ± 18.6	3.3 ± 8.2	−20.6	< 0.0001
Certainty	48.6 ± 30.7	9.6 ± 18.6	−39.0	< 0.0001

*Results shown are based on 37 youth and 53 parent participants who had complete data at T1 (pre-DC) and T3 (10–14 days post-DC) ^1^ Total score from the Decisional Conflict Scale (possible range 0–100). Decreased scores indicate lower decisional conflict and subscale scores

### Choice predisposition / preferred choice

Prior to decision coaching, 73.3% of youth (33/45) and 60.6% of parents (40/66) were leaning towards insulin pump therapy (Table 3), while 20% of youth (9/45) and 35% of parents (23/66) were undecided. One youth and no parents preferred multiple daily injections, before or after decision coaching. After decision coaching, 22% (10/45) of youth and 24% (16/66) of parents reported being undecided. Sixty percent of youth (6/10) and 25% of parents (4/16) expressed a preference prior to coaching. Of those undecided before coaching, 55% (5/9) youth and 48% (11/23) parents chose pump therapy, the others remained undecided after coaching.

**Table 3:** Change in choice predisposition after decision coaching

Pre-coaching choice (T1)	Post-coaching choice (T2)			
	Conventional^a^	MDI^b^	Insulin pump	Undecided
Youth *n* = 45				
Conventional (*n* = 2)	1	0	0	1
MDI (*n* = 1)	0	1	0	0
Insulin pump (*n* = 33)	0	2	26	5
Undecided (*n* = 9)	0	0	5	4
Parents n = 66				
Conventional (*n* = 3)	2	0	1	0
MDI (*n* = 0)	0	0	0	0
Insulin pump (*n* = 40)	2	1	33	4
Undecided (*n* = 23)	0	0	11	12

^a^ Conventional insulin therapy

^b^ *MDI* multiple daily injections

### Agreement about the preferred option

Agreement about the treatment option increased between youth and mother (*n* = 40) from 0.18 prior to decision coaching to 0.51 after coaching. Agreement between youth and father (*n* = 27) increased, from 0.17 pre-coaching to 0.44 post-coaching. Between mother and father (*n* = 22), agreement decreased from 0.66 pre-coaching to 0.48 post-coaching. Agreement between either two (youth and mother or youth and father) (*n* = 45) or three (youth and both parents) (*n* = 22) family members increased from 0.18 pre-coaching to 0.27 post-coaching.

### Participants’ satisfaction with the intervention

Coaching sessions averaged 54.8 ± 8.8 min (range: 30–75 min). Most youth (57%) and parents (90%) rated the length of decision coaching as “just about right” (Table 4). Few youth (8%) and parents (4%) rated the session as ‘much too long’. Almost all youth (92%) and parents (96%) thought that decision coaching and the decision aid helped them to consider their options in a balanced way, while 92% of youth and 91% of parents thought that the shared decision making intervention was ‘somewhat helpful’ or ‘very helpful’ in aiding them come to a preferred option. Most or all participants would definitely or probably recommend decision coaching and the decision aid to others facing an insulin delivery decision (97% youth; 100% parents) or a different health decision (100% youth; 98% parents).

**Table 4:** Acceptability of decision coaching with a patient decision aid

	Response categories	Youth (*n* = 37) Frequency (%)	Parents (*n* = 53) Frequency (%)
How would you rate the length of the decision coaching session? (Mean: 54.8 ± 8.8 min)	Much too short	0 (0)	0 (0)
	A little too short	0 (0)	1 (1.9)
	Just about right	21 (56.8)	48 (90.5)
	A little too long	13 (35.1)	2 (3.8)
	Much too long	3 (8.1)	2 (3.8)
Did the decision coaching help you consider options in a balanced way?	Yes	34 (91.9)	51 (96.2)
	No	0 (0)	0 (0)
	Missing response	3 (8.1)	2 (3.8)
How helpful was the decision coaching in helping you come to a preferred option?	Not helpful	0 (0)	1 (1.9)
	A little helpful	3 (8.1)	4 (7.6)
	Somewhat helpful	12 (32.4)	18 (34.0)
	Very Helpful	22 (59.5)	30 (56.6)
Did the decision coach suggest or recommend a specific option to influence your decision?	Yes	11 (29.7)	5 (9.4)
	No	25 (67.6)	48 (90.6)
	Missing response	1 (2.7)	0 (0)
Would you recommend decision coaching to other people who are facing the same decision?	I would definitely not recommend it	1 (2.7)	0 (0)
	I would probably not recommend it	0 (0)	0 (0)
	I would probably recommend it	12 (32.4)	17 (32.1)
	I would definitely recommend it	24 (64.9)	36 (67.9)

## Discussion

We evaluated an intervention to facilitate shared decision making in youth and parents considering a change in insulin delivery regimens. Overall, we found that youths’ and parents’ decisional conflict decreased after they were involved with decision coaching with a patient decision aid. Youth-parent dyad agreement about the preferred insulin delivery choice also improved after the coaching session. All participants were generally satisfied with the decision support intervention. Our results lead us to make the following observations.

First, healthcare systems are increasingly challenged to partner with patients to achieve patient- and family-centered care [25]. In pediatrics, patient- and family-centered care is described as a mutually beneficial partnership among children, family members, and healthcare professionals [26]. Children and youth have a right to self-determination and are capable of engaging in the decision making process in a manner that is appropriate for their developmental level and experience with their condition. However, few interventions to facilitate pediatric involvement in shared decision making have targeted both children/youth and parents together, and none involved youth with T1D and their parents [10, 11]. Our decision coaching with a patient decision aid intervention promoted collaboration between youth with T1D and their parents by structuring the decision making process to facilitate communication exchange, youth/parent preference elicitation, and support in addressing decision making needs. It empowered youth by inviting them to participate, providing information tailored to their health literacy needs, and encouraging them to speak first when working through the decision making process. Gabe’s theoretical analysis of shared decision making in pediatrics calls for partnership among stakeholders by forming coalitions in decision making to reconcile various perspectives [26]. Indeed, our findings demonstrated improved agreement between youth-parent dyads about the preferred treatment option after engaging in decision coaching.

Second, our decisional conflict results are consistent with the theoretical and shared decision making literature. The Ottawa Decision Support Framework asserts that individuals’ decisional needs affect the quality of a decision (i.e. informed, values-based choices), which in turn affects behaviour (e.g. delaying or changing decisions), health outcomes, emotions (e.g. regret, blame), and use of health services [14]. For example, when patients are more involved in decision making, they are less likely to experience decisional conflict [13, 27]. Similarly, our study noted that after exposure to the shared decision making intervention, there was a decrease in decisional conflict for the youth and parents for the total score and in the subscales of feeling informed, having clear values, feeling supported, and being sure about their decision. Final DCS scores indicate very strong likelihood of following through with the chosen option [21]. The positive impact of our intervention on decisional conflict for youth and parents suggests there may be benefits for their future diabetes management and quality of life.

Third, both parents and youth were generally satisfied with the decision support intervention. However, 8% of youth reported that the median 55-min (range 30 to 75 min) decision coaching sessions were much too long. The length of the decision coaching session in this study was slightly longer than in our pilot study (median 45 min; range 20 to 90) which had 7 youth/parent sessions [12]. Our decision coaching session was specifically designed to engage the youth and the parent(s) as partners in the decision making process. Nonetheless, our results suggest that youth might benefit from re-structuring the way that the intervention is provided. For example, youth might prefer to review or fill in the patient decision aid prior to seeing the decision coach (e.g., in the waiting room or at home) to reduce time spent in the session. However, this may lead to parents influencing the youths’ perspectives if parents encourage or assist the youth in completing it.

Fourth, our study focussed specifically on the preference-sensitive decision about insulin delivery method. There are, however, related decisions that youth with T1D and parents face, such as which insulin pump to choose, which long acting insulin or device, and whether to use continuous glucose monitoring with the chosen insulin delivery method. These decisions can be challenging for youth and families who report insufficient knowledge and uncertainty about their preferences [28, 29]. Although not evaluated in this study, our group is extending the findings of this study to these other preference-sensitive decisions in T1D.

There are four limitations of this study. First, this was not a randomized controlled trial. Instead a pre/post study design with a small sample size was used without a control arm, which could limit observer effect. However, there were significant differences in the primary outcome, the validated decisional conflict scale and its four subscales, for the youth and their parents. Second, our instruments and outcomes were based on self-report, thus inherently subject to social bias. Objective measures (e.g., videotaped consultations) would have allowed for more rigorous evaluation of intervention fidelity and its associated effects on outcomes. Our decision support intervention of decision coaching with a patient decision aid, did not allow us to evaluate these intervention components separately. Self-selection bias may have influenced our findings if families who agreed to participate differed from those who did not. Third, the study group was drawn from our clinic population, which includes urban and rural families, from variable SES, ethnicities and racial groups. However, we are not able to determine how participating parents’ age, marital status or level of education compares to that in our diabetes clinic as that information is not collected in our clinic. It is noteworthy that at the time of our study, the preference in favour of insulin pump therapy was the same in our clinic population as it was amongst study participants. Before and after coaching, a very high proportion of youth and parents expressed preference for pump therapy over multiple daily injections or standard therapy, although after coaching there was movement in both groups to and from a preference for pump therapy. At the time of the study, MDI was rarely used by children or youth in our centre. Since then, MDI has become the usual insulin delivery method from diagnosis onwards. It would be interesting to see how this study’s results might change if conducted again in the current climate in our clinic given that MDI has become the usual insulin delivery method for children and youth. Fourth, the patient decision aid used in this study was based on national clinical practice guidelines evidence and the clinical experience of our site’s pediatric diabetes health professionals rather than a de novo systematic review. This may have led to incomplete or biased evidence in development of our tool. Nevertheless, the coaching and decision aid were well received by youth and families who recommended their use with others facing the same or other decisions.

## Conclusion

Our decision support intervention using decision coaching with a patient decision aid promoted shared decision making between youth and parents who were considering whether to change the youths’ insulin delivery method. This approach used a structured process to minimize power imbalances between stakeholders when discussing the treatment options. The results showed reductions in youth and parent decisional conflict, with good satisfaction amongst youth and parents. Given that decision coaching with patient decision aids for youth and parents is relatively novel, future research that evaluates the efficacy and effectiveness of such interventions using experimental methods (e.g., randomized controlled trial) is required. However, given the strong evidence for patient decision aids in adults, it is unethical to randomize youth and parents to usual care. Hence, our clinic routinely uses decision coaching with a patient decision aid to support youth and their family facing decisions about insulin delivery options.

## Data Availability

Our research ethics board did not approve public access of our raw data. However, our data could be potentially publically available to researchers upon a reasonable request and if satisfactory permissions are obtained.

## Abbreviations

DCS: Decisional Conflict Scale
OFDG: Ottawa Family Decision Guide
T1D: Type 1 diabetes
